# Periplasmic Expression of TNF Related Apoptosis Inducing Ligand (TRAIL) in *E.coli*

**Published:** 2015

**Authors:** Omid Tavallaei, Mojgan Bandehpour, Nastaran Nafissi-Varcheh, Bahram Kazemi

**Affiliations:** a Department of Pharmaceutical Biotechnology, School of Pharmacy, Shahid Beheshti University of Medical Sciences, Tehran, Iran.; b Cellular and Molecular Biology Research Center, Shahid Beheshti University of Medical Sciences, Tehran, Iran.; c Biotechnology Department, School of Medicine, Shahid Beheshti University of Medical Sciences, Tehran, Iran.

**Keywords:** TRAIL, Periplasmic expression, *E. coli*, Signal sequence, Secretion

## Abstract

Tumor necrosis factor-related apoptosis-inducing ligand (TRAIL), a member of TNF family, is an interesting ligand which selectively induces apoptosis in tumor cells and, therefore, it has been developed for cancer therapy. This ligand has been produced by various hosts such as *E.coli*. However, protein expression in *E.coli* cytoplasm leads to problems such as incorrect folding, reduction in biological activity, inclusion body formation, and sophisticated downstream. The aim of this study is to develop an expression system for the production of recombinant TRAIL secreted to the *E.coli* periplasm instead of cytoplasm. By using Overlapping Extension PCR, an OmpA signal sequence was fused to TRAIL cDNA and OmpA-TRAIL fragment was then cloned in pET-22b plasmid. This construct was confirmed by PCR and DNA sequencing. Promoter was induced in *E.coli* BL21 (DE3) and periplasmic expressed proteins were released using osmotic shock procedure. SDS-PAGE analysis showed that about 37% of recombinant TRAIL was transferred into the periplasm and its identity was confirmed by western blot analysis. Finally, the cytotoxic activity of TRAIL against HeLa cell line was confirmed by using MTT assay. The results demonstrate that our expression system may be useful for the production of TRAIL in the periplasmic space.

## Introduction

Tumor necrosis factor-related apoptosis-inducing ligand (TRAIL) is a potent cytotoxic ligand belonging to the TNF family (1). TRAIL presents significant advantages in comparison with other TNF family members such as TNF-α and Fas Ligand (FasL) (2). Unlike other members, TRAIL induces apoptosis only in tumor cell lines with minimal cytotoxicity against normal cell lines *in-vitro* as well as *in-vivo*, for example, TRAIL has not shown any detectable cytotoxicity in animal models such as murine (3) and primate (4). Also, most tissues can be targets for TRAIL because its receptors are widely expressed in most tissues and cell types (5). Therefore, TRAIL is currently being developed as a cancer therapeutic agent (6).

Several expression systems have been used for the production of recombinant TRAIL such as *E.coli* (7), *P.pastoris* (8), Sf9 insect cells (9), and CHO cells (10). Of these expression systems, *E.coli* is the most frequently used host for the production of TRAIL due to its safety, simplicity, low cultivation cost, and known genetic properties (11).

Although there are a number of reports on the cytoplasmic expression of recombinant TRAIL in *E.coli*, its expression in the *E.coli* cytoplasm represents some problems (7, -). Firstly, over-expression of TRAIL in the cytoplasm leads to the formation of inclusion bodies. The isolation of target protein from inclusion bodies requires several refolding steps in order to obtain soluble and biologically active protein which results in process complexity and yield reduction (15). Secondly, several purification steps are required to purify the protein of interest among a large number of contaminating proteins in the cytoplasm. Finally, the expression yield may be low due to the high protease activity of the cytoplasmic compartment (16). 

Directing the expressed TRAIL into the periplasmic space using a signal sequence can be an approach to resolve the above-mentioned problems and periplasmic protein can be easily isolated by selective disruption of the *E. coli* outer membrane resulting in a reduction in purification steps, complexity, cost, and time. In addition, TRAIL can be properly folded in periplasm which means it does not require the time-consuming refolding processes to have biological activity (16, 17).

Secretory prokaryote proteins which are transported through cytoplasmic membrane typically contain an N-terminal signal sequence. Outer Membrane Protein A (OmpA), a major outer membrane protein of *E.coli*, contains an N-terminal signal sequence which has frequently been used for periplasmic expression of recombinant proteins in *E.coli*. The OmpA signal sequence interacts with Sec-dependent secretion pathway agents. In this pathway, the cytoplasmic chaperone SecB binds an unfolded pre-protein harboring the OmpA N-terminal signal sequence and transfers them to inner membrane-bound SecA. Subsequently, the pre-protein crosses the inner membrane through SecYEG channel and the signal sequence is cleaved by a signal peptidase (-).

 The purpose of the present study was to investigate the periplasmic expression of TRAIL in *E.coli* using a designed vector based on pET-22b plasmid, a frequently used expression vector, and the OmpA signal sequence (19).

## Experimental

*Construction of expression vector*

**Figure 1:**
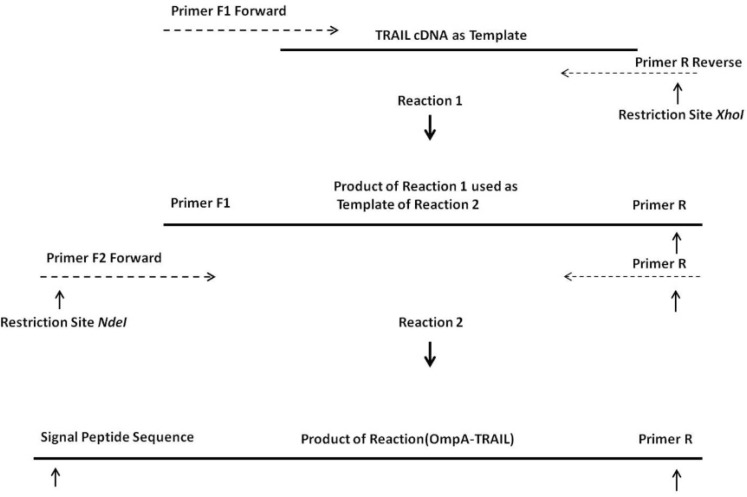
OE-PCR protocol for the insertion of the OmpA signal sequence into the TRAIL cDNA. Reaction 1: hybridization of 3´ region of primers F1 with terminal regions of TRAIL cDNA and synthesis a part of the OmpA signal sequence. Reaction 2: using the product of the reaction 1 as a template by primer F2 and completion of whole OmpA signal sequence.

Overlapping Extension Polymerase Chain Reaction (OE-PCR) method was used in order to insert OmpA signal sequence into the 5´ region of human TRAIL cDNA, which had been previously prepared in our laboratory (20). For this purpose, DNA encoding OmpA signal sequence was added at 5' end of TRAIL cDNA through two cycles of PCR using pfu DNA polymerase (Fermentas, Lithuania) (Figure 1). In the first run of PCR, a primer pair was partially annealed to TRAIL cDNA with overlapping end region. During PCR reaction, the overhang region of the forward primer, which is a part of OmpA signal sequence, was inserted at 5' end of DNA template. On the other hand, *Xho*I restriction site was introduced at 3' end of DNA template. The first PCR product was used as a template for the second run and the second forward primer was partially annealed with 5' end region of this template resulting in the synthesis of full-length OmpA signal sequence and *Nde*I restriction site at 5' end of TRAIL cDNA. 

Two forward primers and one reverse primer (MWG, Germany) applied for OE-PCR was shown as Table 1. The forward primer for the first PCR (F1) contained a 3' overlapping region with 5' end of TRAIL cDNA. Likewise, the forward primer of the second PCR (F2) contained a 3' overlapping end region with 5' end of primer F1 thereby enabling the F2 primer to anneal with the first PCR product. The same reverse primer (R) was used in both PCR reactions.

**Table 1:** Primers applied for the insertion of OmpA signal sequence into the 5´ region of TRAIL cDNA.

aPrimer name	bSequence	Restriction site
F1	5´ TGGCACTGGCAGGTTTTGCTACAGTTGCGCAGGCAGTGAGAGAAAGAGGTCCTCAGAGAG 3´	-
F2	5´ AAAAAA*CATATG*AAAAAAACAGCGATTGCGATTGCGGTGGCACTGGCAGGTTTTGC 3´	*Nde*I
R	5´ AAAAAA*CTCGAG*TCCGCGTCCAACTAAAAAGGCCCCGAA 3´	*Xho*I

a : F = Forward R = Reverse

b : Highlighted nucleotides of the F1 and F2 primers are similar to the 5´ end region of the TRAIL and F1 primer, respectively. Highlighted nucleotides of R primer are similar to the 3´ end region of TRAIL. Six Adenine nucleotides included in 5´ end of primers F2 and R were designed in order to provide their recognition by the restriction enzymes.

Each PCR reaction was performed under the following conditions: pre-pre-denaturation at 94 °C for 2 min; first 5 cycles of 94 °C for 30 s (denaturation), 65 °C for 30 s (annealing), and 72 °C for 2 min (extension); next 30 cycles of 94°C for 30 s, 69 °C for 30 s, and 72 °C for 2 min; and post-extension by 72 °C for 5 min. Note that latter 30 cycles differ from former 5 cycles in annealing temperature. 

**Figure 2:**
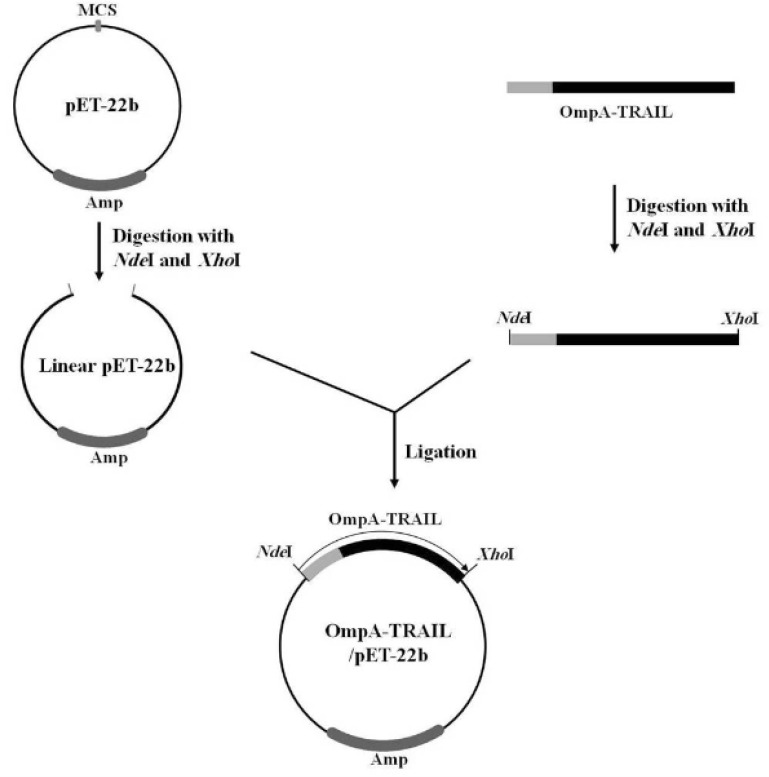
Cloning of OmpA-TRAIL fragment in pET-22b expression plasmid. OmpA-TRAIL fragment and pET-22b plasmid were digested separately with restriction Enzymes *Nde*I and *Xho*I. Then, digested fragment and plasmid were ligated using T4 DNA ligase.

In the next step, the PCR product was purified and digested with *Nde*I and *Xho*I (Fermentas, Lithuania) and then ligated to the corresponding sites of the expression vector pET22b (Novagen, U.S) using T4 DNA ligase (Fermentas, Lithuania) (Figure 2). The resulting plasmid was named OmpA-TRAIL/pET22b and the correct cloning was confirmed by PCR and sequencing. In this expression system, the TRAIL is expressed as fused to a C-terminal poly-histidine tag, thereby making a simple one-step purification of the fusion protein by Ni-NTA affinity chromatography possible.

*Expression of TRAIL and cell fractionation*

The recombinant plasmid OmpA-TRAIL/pET22b was transformed into the *E.coli* BL21 (DE3) by treatment with 0.1 M cold CaCl_2 _and then heat shock at 42 °C for 1 minute_. _The transformed bacteria was cultured in Terrific Broth (TB) medium (1.2% peptone; 2.4% yeast extract; 72 mM K_2_HPO_4_; 17 mM KH_2_PO_4_; and 0.4% glycerol; pH 7.2) on a rotary shaker (200 rpm) at 37 °C until the optical density at 600 nm (OD600) of the culture reached 0.6. Then, isopropyl-β-D-thiogalacto-pyran-oside (IPTG) was added to a final concentration of 0.1 mM to induce the expression of the target protein until 24 h.

Subsequently, cultures were separated into periplasmic and cytoplasmic fractions according to the method of Khosla and Bailey (21). Briefly, 2 mL of culture was centrifuged for 5 minutes at 15,000×g and the pellet was resuspended in 2 mL of a hypertonic sucrose solution (0.2 M Tris; 200 g/L sucrose; 0.1M EDTA; pH 8.0). After shaking incubation for 20 min, cells were harvested by centrifugation for 15 minutes at 15,000×g and resuspended in 2 mL of a second solution (10 mM Tris; 5 mM MgSO_4_; pH 8.0) followed by shaking incubation on ice for 10 minutes. The resulting spheroplasts (*i.e*. cells without outer membrane) were centrifuged for 10 minutes at 5000×g at 4 °C and the supernatant was kept as the periplasmic fraction. Spheroplasts were resuspended in 2 mL of the second solution and disrupted by ultrasonication in cycles of 20 s sonication and 20 s chilling on ice (dr.hielscher, Germany). The cycles were repeated until the solution was transparent. Cell debris was removed by centrifugation (10 min; 15,000×g) and the supernatant was kept as the cytoplasmic fraction.

*SDS-PAGE and western blotting analysis*

SDS-PAGE was performed in a 12% resolving polyacrylamide gel according to the Laemmli method (22) and 10 µg of proteins were loaded in each lane. For immunoblotting, proteins were resolved on a 12% polyacrylamide gel and then transferred to a nitrocellulose membrane (Whatman, U.S). After washing two times for 5 minutes with TBS (10 mM Tris; 150 mM NaCl; pH 8.0), the membrane was blocked with 3% non-fat milk in TBS for 1h and subsequently washed three times for 5 minutes with TBST (TBS containing 0.05% Tween 20). The membrane was incubated with 1:1000 anti-TRAIL antibody (Abcam, U.K) at room temperature for 2 h. After washing three times for 10 minutes with TBST, the membrane was incubated with 1:1000 Alkaline phosphatase conjugated secondary antibody (Abcam, U.K) at room temperature for 2 h. Western blot was developed using the BCIP/NBT substrate system (Sigma–Aldrich, Germany).

*Purification of recombinant protein*

The periplasmic fraction containing TRAIL was purified by Ni-NTA affinity chromatography. For this purpose, 2 mL of the 50% Ni-NTA His-bind resin slurry charged with Ni^2+^ (Novagen, U.S) was equilibrated with the binding buffer (50 mM NaH_2_PO_4_; 300 mM NaCl; 10 mM imidazole; pH 8). The periplasmic fraction solution was loaded onto the column and washed two times with 4 mL of washing buffer (50 mM NaH_2_PO_4_; 300 mM NaCl; 20 mM imidazole; pH 8). Bound protein was eluted four times with 0.5 mL of eluting buffer (50 mM NaH_2_PO_4_; 300 mM NaCl; 500 mM imidazole; pH 8). Elution fractions were pooled and dialyzed against PBS buffer using a 12 KDa cut-off membrane. Then, protein solution was concentrated by dialysis against sucrose powder which draws water through the membrane. Finally, 1 µg of purified protein solution and 10 µg of periplasmic fraction were subjected to analysis by SDS–PAGE. Protein concentrations were measured based on absorbance at 280 nm using a nanodrop spectrophotometer (Eppendorf, Germany).

*Biological activity of TRAIL*

Human cervical cancer HeLa cells (Pasteur Institute, Iran) were used to assess the cytotoxicity of expressed TRAIL and dispensed in 96-well cell culture plates (2×10^3^ cells/well). As reported before (7), different concentrations of purified periplasmic TRAIL (1, 4, 8, 16 mg/L) were added to each well. It had been previously observed that 24 h incubation time is more favorable for detecting cell cytotoxicity (data not shown). Therefore, the plate was incubated at 37 °C, 5% CO_2_ for 24 h. The culture medium was Roswell Park Memorial Institute (RPMI) medium containing 10% Fetal Calf Serum (FCS), 100 U/mL penicillin and 100 µg/mL streptomycin. Following the incubation, cytotoxicity was assessed by the MTT protocol as previously described (23).

## Results and Discussion

*Construction of expression vector*

**Figure 3:**
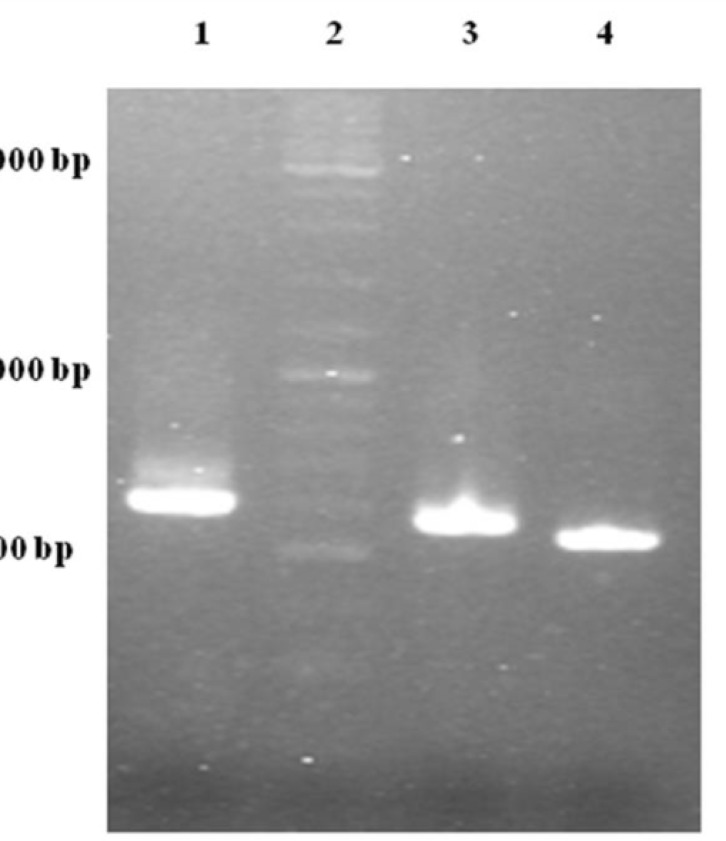
Gel electrophoresis analysis of OE-PCR products. Lane 1: 594 bp product amplified with F2R primer indicating OmpA-TRAIL whole sequence. Lane 2: DNA Ladder marker. Lane 3: 557 bp product amplified with F1R primer indicating partially-synthesized signal sequence. Lane 4: 504 bp product amplified with TRAIL specific primers indicating TRAIL sequence.

One of the most important elements for transporting a recombinant protein into the periplasmic space is an N-terminal signal sequence (24). For this purpose, an OmpA signal sequence was fused to the 5´ region of the TRAIL cDNA using two sequential OE-PCR reactions (Figure 1). The OmpA signal sequence was chosen because it functions efficiently to secrete a large amount of the OmpA protein (25) and it is frequently used in several studies attempting to secrete different proteins (-). As expected, the size of the PCR products sequentially increased from 504 bp to 594 bp indicating a correct fusion of the OmpA signal sequence to TRAIL cDNA (Figure 3). 

The increase in size of the products after each PCR reaction demonstrates that the 5´ region of the TRAIL fragment is extended due to the primer design (Table 1). In the first cycles of each PCR reaction, the forward primer was hybridized with 5´ region of template by 19-25 overlapping nucleotides of its own 3´ region. Then, in the later cycles, the overhang sequence of the forward primer was inserted into the template gradually. Therefore, in the first 5 cycles, annealing temperature was considered 65°C according to the overlapping region, whereas in the next 30 cycles it was considered 69°C according to the whole primer sequence. In addition, a high-fidelity DNA polymerase pfu was used to ensure that the rate of mutations in sequential PCR reactions was minimal.

Finally, in order to express recombinant TRAIL, the PCR product was purified, digested with *Nde*I and *Xho*I, and then sub-cloned into the corresponding sites of the pET-22b plasmid. The correct insertion of OmpA-TRAIL into the recombinant plasmid was confirmed by PCR analysis and DNA sequencing. 

*Expression and purification of recombinant TRAIL*

The ability of recombinant plasmid OmpA-TRAIL/pET22b to express and transport the recombinant protein into the periplasmic space was analyzed by expression induction and then isolation of periplasmic and cytoplasmic proteins. The recombinant plasmid was transformed into the *E.coli* BL21 (DE3) and cultivated in TB culture medium followed by induction of TRAIL expression with 0.1 mM IPTG. In order to release periplasmic proteins, outer membrane was selectively disrupted by osmotic shock procedure. Cytoplasmic proteins were then released by disruption of resulted spheroplasts. Whole cells were also disrupted to assess the total expression of TRAIL. 

**Figure 4:**
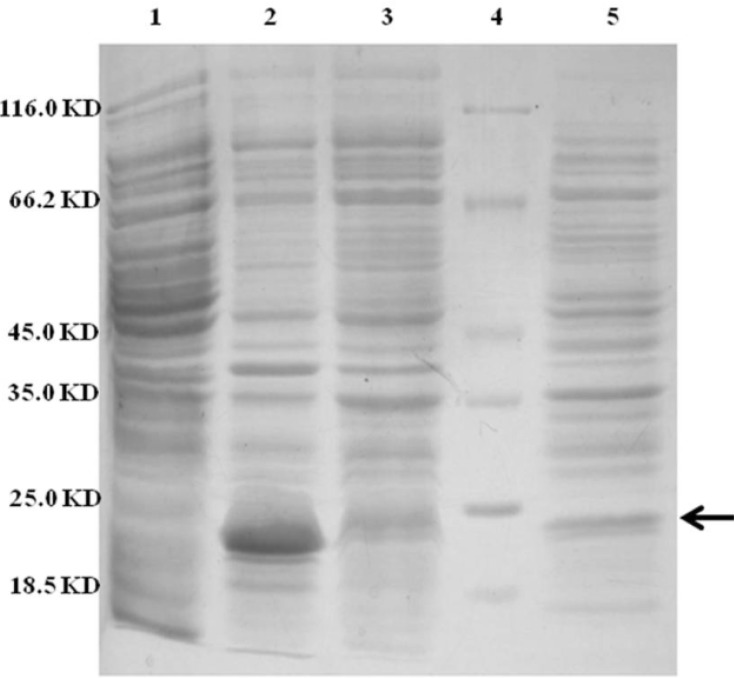
SDS-PAGE analysis of different cellular fractions of *E.coli* BL21(DE3) including OmpA-TRAIL. Lane1 and 2: whole cell before and after induction, respectively. Lane 3: cytoplasmic fraction after induction. Lane 4: protein marker. Lane 5: periplasmic fraction after induction. 10 µg of proteins were loaded in each lane.

Approximately 37% of total expressed TRAIL was secreted into the periplasmic space based on measuring the staining intensity of protein bands using ImageJ software (NIH) (Figure 4). The results are consistent with the recent report of Sockolosky *et al*. (26) showing that the recombinant human growth hormone is directed to the *E. coli* periplasm using the pET based expression based on OmpA signal sequence. Also, several studies have shown the efficacy of the OmpA signal sequence to direct recombinant proteins into the periplasmic space (29, 30)

However, although the level of TRAIL secretion into periplasm was satisfactory, it seems that a considerable amount of recombinant TRAIL was remained in *E.coli* cytoplasm. It might be explained by a rather high expression rate and, therefore, lack of time for interaction between the signal sequence and the transport pathway agents leading to remaining target protein in the cytoplasm as inclusion bodies. (31). Low concentration of IPTG (0.1 mM) was applied in order to control the expression, but high cultivation temperature (37 ºC) may be responsible for high expression rate. (16, 32). On the other hand, secretion into the periplasm is a complex process and the presence of a signal sequence does not always ensure the efficient protein secretion (18). 

Subsequently, periplasmic TRAIL was purified for further study on its biological activity. Cloning in vector pET-22b fuses a histag to the C-terminal of expressed protein which was used for purification of periplasmic TRAIL by Ni-NTA affinity chromatography. 

**Figure 5:**
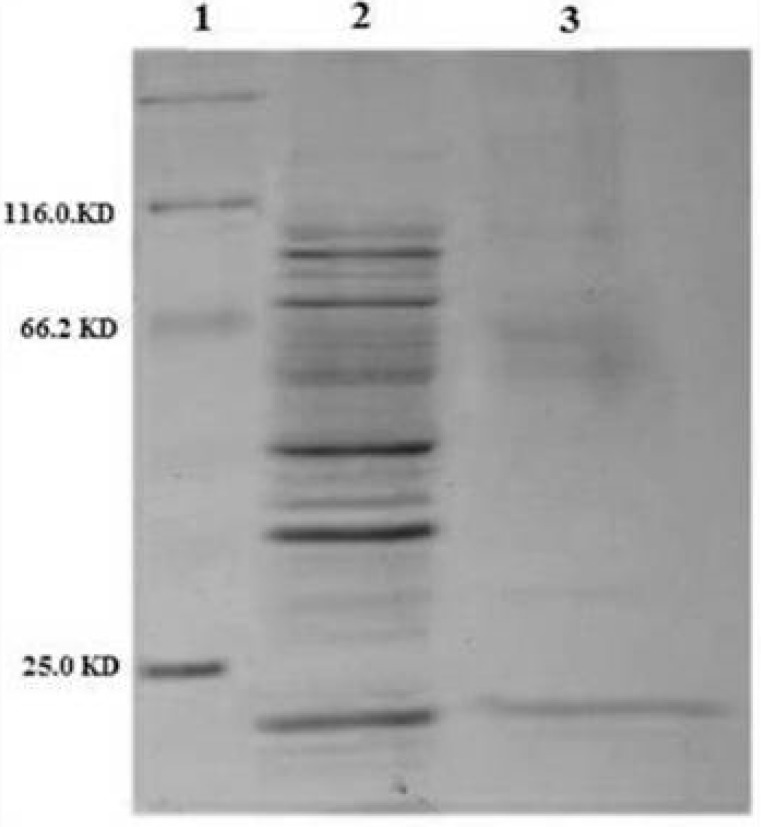
SDS-PAGE analysis of purified periplasmic TRAIL by Ni-NTA affinity chromatography. Lane 1: protein marker. Lane 2: 10 µg of periplasmic fraction of *E.coli* BL21 (DE3) after induction. Lane 3: 1 µg of purified TRAIL after elution by imidazole

Figure 5 shows an efficient purification of the periplasmic TRAIL in which other contaminating proteins were mostly removed. The obtained results of purification study were summarized in Table 2.

 Eluting buffer has some components such as imidazole which interfere with the assessment of biological activity of TRAIL (33). For this reason, eluting fractions were dialyzed against PBS buffer to remove any interfering components.

**Table 2:** Purification of periplasmic TRAIL from *E*.coli.

**Sample**	**Volume (mL)**	**Total Periplasmic Protein (mg)** a	**Periplasmic TRAIL (mg) ** b	**Purity (%)**	**Purification Yield (%)**
Culture	100	5	0.78	16	-
Purified Solution	1	0.75	0.68	91	87

a Protein concentration was determined based on absorbance at 280 nm.

b TRAIL concentration and purity were estimated by scanning the SDS-PAGE gel using ImageJ software.

*Immunological identification of recombinant TRAIL*

**Figure 6:**
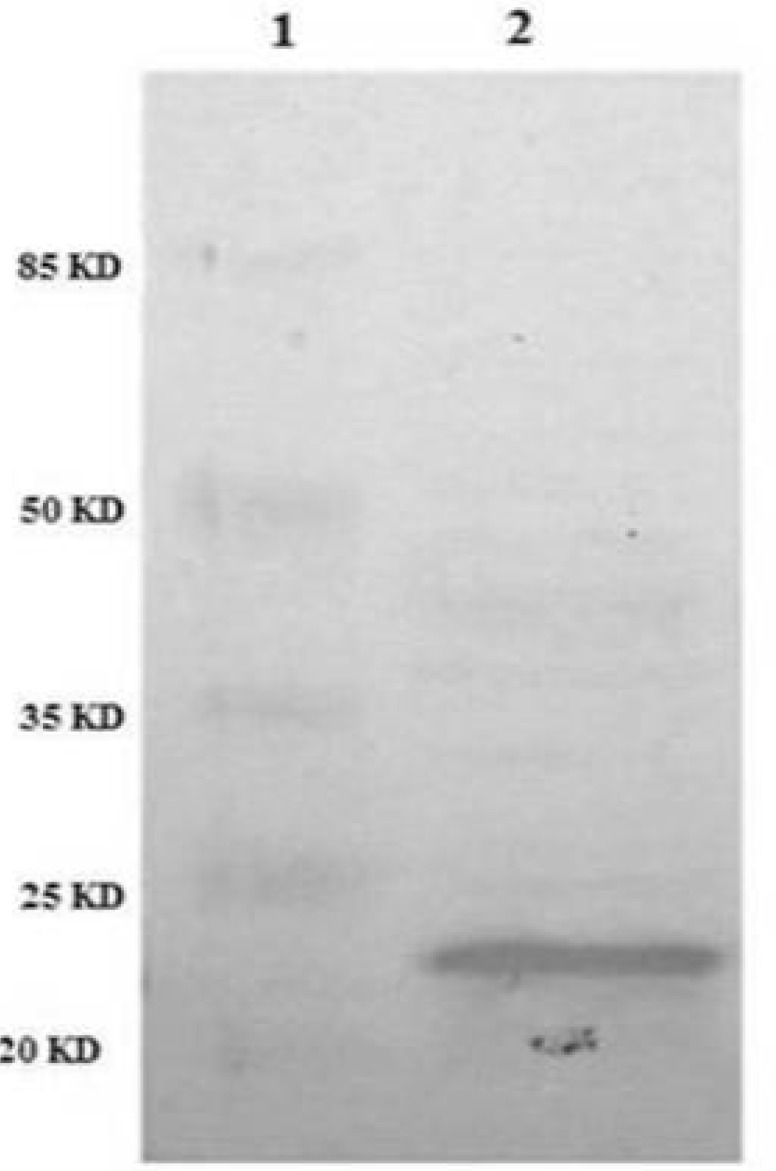
Identification of periplasmic TRAIL protein by Western Blot. TRAIL was resolved by SDS-PAGE and then transferred to nitrocellulose membrane. After incubation with anti-TRAIL antibody and then secondary antibody, the membrane was developed with the BCIP/NBT substrate system. Lane 1: protein marker. Lane 2: periplasmic TRAIL.

In order to confirm the identity of recombinant TRAIL immunologically, western blot analysis was performed using anti-TRAIL antibody. The results showed that periplasmic fraction reacted with anti-TRAIL antibody and the size of the protein was approximately 21 kD as expected for recombinant TRAIL (Figure 6).

*Biological activity of TRAIL*

**Figure 7:**
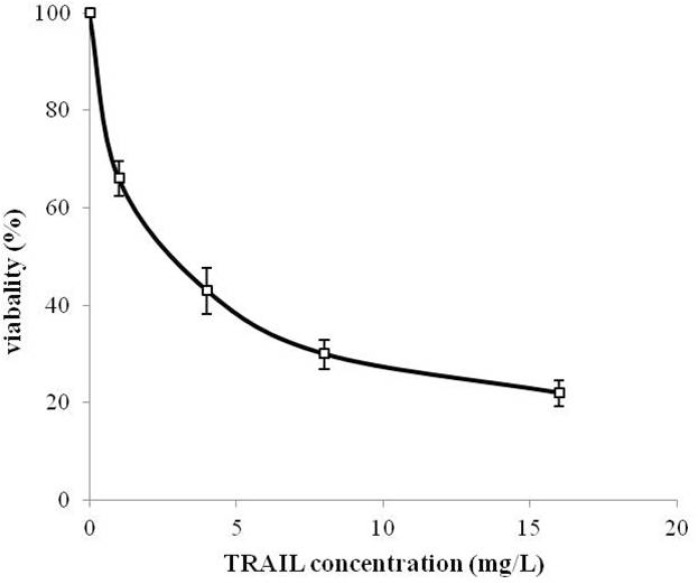
Cytotoxicity of periplasmic expressed TRAIL in human cervical cancer HeLa cells. HeLa cells were dispensed into 96-well plate (2×10^3^ cells/well) and treated with different concentrations of TRAIL for 24 h. Cytotoxicity was then assessed based on MTT assay protocol. All measurements are reported as mean ± SD (n=3).

MTT assay protocol was performed to evaluate the cytotoxicity effect of periplasmic expressed TRAIL against human cervical cancer HeLa cells. HeLa is one of the most frequently used cell lines in pharmaceutical research studies for assessment the anti-tumor activity (34, 35). Periplasmic expressed TRAIL induced cytotoxicity in HeLa cells in a dose-dependent manner (Figure 7). The cytotoxicity was detectable for concentration of 200 µg/L of periplasmic TRAIL. Also, the ED_50_ was about 2.7 mg/L determined by GraphPad Prism software (version 6.0, Graphpad software). This cytotoxicity effect was in accordance with the previous studies (7, 31). However, in those studies, recombinant TRAIL was expressed as inclusion body in *E.coli* and needed to be refolded before assessing its cytotoxic effect, while, in the present study, periplasmic TRAIL was soluble and biologically active. Therefore, it could be concluded that TRAIL directed to the periplasm has a proper folding. In other expression systems (*e.g*. *P.pastoris*), recombinant TRAIL has shown cytotoxic effect against various cancer cell lines (-). However, it seems that efficiency of TRAIL cytotoxic effect may partially be varied depending on cancer cell lines, expression systems, and source of recombinant TRAIL (*i.e*. human or animal) (36).

In this paper a simple method for production of the recombinant TRAIL directed to the *E. coli* periplasm is presented using pET expression system and OmpA signal sequence. The results demonstrated efficient periplasmic expression of TRAIL. Considering the advantages of directing recombinant TRAIL into the *E.coli* periplasmic space, this study may be useful for production of this therapeutic ligand in *E.coli* periplasm.
